# Anthropometrics of neonates born to mothers with diabetes in pregnancy in the Northern Territory

**DOI:** 10.1186/1687-9856-2015-S1-P111

**Published:** 2015-04-28

**Authors:** Danielle Longmore, Alex Brown, I-Lynn Lee, Christine Connors, Cherie Whitbread, Marie Kirkwood, Jeremy Oats, David McIntyre, Jonathan Shaw, Paul Zimmet, Kerin O’Dea, Louise Maple-Brown

**Affiliations:** 1 Menzies School of Health Research, Darwin, NT, Australia; 2 South Australian Health and Medical Research Institute, Adelaide, SA, Australia; 3 Northern Territory Department of Health, Darwin, NT, Australia; 4 Melbourne School of Population and Global Health, Melbourne, VIC, Australia; 5 Mater Medical Research Institute, Brisbane, QLD, Australia; 6 Baker IDI Heart and Diabetes Institute, Melbourne, VIC, Australia; 7 University of South Australia, Adelaide, SA, Australia

## 

Type 2 diabetes (T2DM) is increasing in prevalence in Indigenous Australian children and adolescents. High rates of diabetes in pregnancy (DIP) in Indigenous Australians increases the risk of diabetes for the next generation. DIP is associated with neonatal adiposity, which correlates with long-term risk of obesity and diabetes. Indigenous Australians have high rates of low birth weight and increasingly, large for gestational age associated with DIP.

The aims are: 1. To evaluate adiposity in babies born to Indigenous mothers and those of European background with DIP in the Northern Territory; 2. To evaluate the relationship between maternal factors and neonatal birth weight and body composition.

Thus far 266 mothers and neonates from the PANDORA cohort (Pregnancy and Neonatal Outcomes in Remote Australia) have been assessed. Neonatal anthropometrics were performed on all neonates, including skin fold measures. Calculations of fat mass were made using a validated equation (fat mass=0.39055(birth weight)+0.0453(flank skinfold)-0.03237(length)+0.54657).

Significant differences were found in maternal characteristics between Indigenous and European background participants, including diabetes type (T2DM 14.7% vs 1.1%, p<0.001), smoking in pregnancy (26.5% vs 9.1%, p<0.001) and location of residence (regional/remote 41.4%vs 9.8% p<0.001). Gestational age at birth was significantly different (38.2 vs 39 weeks p<0.001), however birth weight was not significantly different (3380 vs 3428g). Indigenous neonates had greater subscapular (4.69 vs 4.20mm, p=0.003) triceps (4.75 vs 4.22mm, p=0.004) and flank skin folds (4.08 vs 3.60mm, p=0.006). This difference remained significant for the flank skin fold only, after adjustment for diabetes type and maternal body mass index (BMI). There was no significant difference in calculated fat mass. On regression analysis, maternal BMI, smoking, nulliparity and T2DM were each independently associated with birth-weight z-score.

Recruitment to PANDORA is ongoing. Preliminary data reveals higher skin fold measures, indicative of adiposity, in Indigenous neonates. There was no significant difference in fat mass. Smoking, BMI, nulliparity and T2DM were independently associated with birth-weight z-score, ethnicity was not independently associated.

